# The Impact of a Horse Riding Intervention on the Social Functioning of Children with Autism Spectrum Disorder

**DOI:** 10.3390/ijerph14070776

**Published:** 2017-07-14

**Authors:** Androulla Harris, Joanne M. Williams

**Affiliations:** Clinical and Health Psychology, University of Edinburgh, Edinburgh EH8 9AG, UK; androulla_harris@hotmail.com

**Keywords:** autism spectrum disorder, ASD, animal-assisted intervention, therapeutic horse riding, social functioning, human-animal interaction, children

## Abstract

This paper reports a case-control study of a horse riding intervention for children with autism spectrum disorder (ASD). A sample of 26 children, aged 6 to 9 years, were assigned to either the intervention (*n* = 12) or control group conditions (*n* = 14). Pre- and post-tests were carried out using the Childhood Autism Rating Scale, Second Edition (CARS2) and the Aberrant Behaviour Checklist-Community Edition (ABC-C). An observational measure of compliance and behaviour during the horse riding sessions was completed for the intervention group. There was a significant reduction in the severity of ASD symptoms and hyperactivity from pre- to post-test for the intervention group only. These results indicate that the intervention improves some aspects of social functioning for children with ASD.

## 1. Introduction

There is growing interest in the appropriateness and effectiveness of animal-assisted interventions (AAI) for children with autism spectrum disorder (ASD). Recent systematic reviews [1,2,3,4] have highlighted the evidence for the beneficial impact of AAIs, but also point to the need for further high quality research studies. This paper reports a controlled intervention evaluation of a horse riding intervention for children with ASD.

### 1.1. ASD and AAIs

ASD is a neurodevelopmental disorder with a diagnosis based upon repetitive stereotypic behaviours and impaired abilities in communication and social interaction [5]. The latter may be presented by difficulties establishing and sustaining relationships, a lack of eye contact, and deficiencies in social recipriocity and Theory of Mind (ToM) [6,7]. Symptoms of ASD can be socially disruptive at times, and the general public has limited knowledge or understanding of ASD [8]. Research using US longitudinal data has shown that young adults with ASD are significantly more prone to social isolation than young adults with other intellectual, emotional or behavioural disabilities [9], with negative implications for their quality of life [10].

AAIs may offer an alternative approach to traditional interventions for children with ASD [2,3,4,11]. AAIs include animal-assisted therapies (AAT), which use a trained therapist and therapy animal, and animal-assisted activities which do not [12]. AAIs can involve short-term structured activities or longer-term interaction with an assistance or service animal [13]. They are a type of “Green Care”, connecting people with nature and animals to enhance health and wellbeing [14,15,16,17]. Animals offer non-judgemental calming support [12] leading to a range of social, psychological, physiological and physical benefits [18].

The value of AAIs for children with ASD was first recognised by Levinson in 1964 [19], and since then studies have indicated that animals can help by acting as companions [20] or service animals [21,22]. AAIs have beneficial outcomes for children and adolescents with ASD [20,23,24,25]; specifically, reduced stress, problem behaviours and ASD severity, and increased wellbeing, language and communication [4]. Children and adolescents with ASD display greater social interaction with an interventionist/therapist in the presence of an animal [20,25,26,27]. For example, O’Haire, McKenzie, Beck and Slaughter (2013) [28] found that children with ASD engaged with an adult and typically developing peers more pro-socially in the presence of guinea pigs, rather than toys. Other studies have reported significant increases in measures of social interaction among children with ASD, following an AAI involving horses [23,29,30,31].

AAIs for children with ASD have most commonly involved horses and dogs [1,4]. Horse riding interventions have had positive or mixed outcomes for social skills, communication and behaviour [1,24,29,30,31,32,33,34,35,36]. Although the relative benefits of using different species in AAIs for ASD have not been investigated [4], horses may be particularly well-suited to reducing anxiety in children with ASD. Horses selected as therapy animals typically have calm temperaments [37] and slower, steadier movements than smaller therapy animals. Therefore, horses selected for use in AAIs are less likely to move in a rapid unpredictable way, which children with ASD characteristically dislike [23]. Horses move to a rocking rhythm that can be calming and make sensory stimuli in the immediate surroundings seem less overwhelming. Additionally, since horses are responsive to their rider’s subtle movements and cues, the rider needs awareness and control of their own body movements in order to interact with the horse. Thus, horses may help individuals with ASD to understand their behaviours’ impact in a tangible cause-and-effect way. This is well-suited to their favoured learning style [30]. There is promising evidence that equine interventions may also be effective in treating other chonic illnesses and health challenges [38].

AAIs are an emerging area of research and the psychological mechanisms underpinning their effects are currently debated. Firstly, AAIs may create a “social catalyst” or “social facilitation” effect, where an animal facilitates social interactions between humans [39,40,41,42,43]. This may be achieved through an animal’s presence and spontaneous behaviour providing a neutral focal point between the child and their interventionist/therapist [40]. Secondly, AAIs may increase empathy and understanding of other’s minds, which are both required for social interaction [44]. Indeed, a case study of a child with ASD showed that their empathy levels increased after a service animal was incorporated into their daily life at home [45]. Thirdly, people with ASD may experience less discomfort during social situations with animals [28,46,47]. Animals’ signalling of emotion may be more behaviourally salient than humans’. This may make interactions with animals less socially complex and cognitively demanding [37]. Furthermore, an individual with ASD who fails to interpret subtle communication cues or to comply with social norms [37] will not face judgement or disdain from an animal. Finally, the animal may act as a transitional object [48] which the child forms a bond with, that extends to a bond with a human [49,50].

In addition to possible psychological mechanisms, physiological processes and anxiety reduction might contribute to AAIs’ efficacy. This may be important in the context of young people with ASD, 40% of whom have at least one comorbid Diagnostic and Statistical Manual (DSM)-IV anxiety disorder [51]. Research using physiological data from typically developing children and adults has demonstrated that anxiety can be reduced by the presence of an animal [52] and by positive physical contact with an animal, such as stroking [53]. For children with ASD, decreased stress after participation in an AAI has been demonstrated by one study using physiological data [22], and others using qualitative [54] and observational data [23].

Overall, few studies investigate mechanisms of change in AAIs [55,56]. In fact, May et al. (2016) [3] systematic review of AATs for youth found only 2.2% of studies had tested for mediators. There is also a need for AAI studies to explore how levels of engagement with an intervention (dosage) may relate to treatment effects.

### 1.2. Methodological Issues

AAI for ASD is a relatively new area of research and many existing studies have methodological weaknesses. They provide preliminary support rather than strong evidence that AAIs are beneficial to young people with ASD [1,4]. Limitations include lack of control groups, small samples with weak statistical power, lack of pre-test data, and use of outcome measures and treatment structures that are not comparable across studies [1,2,3,4]. Thus, the replicability and generalisability of their findings can be questioned [55]. Methodological limitations in AAI research may explain why reviews of ASD interventions rarely include AAI research [4], or only include them as “unestablished treatments”.

It should be noted that since the most recent systematic review of AAIs for ASD [1], the first randomised controlled trial (RCT) of therapeutic horse riding for youths with ASD has been published [57]. Participants’ mean age was 10.2 years, and their ages ranged from 6-16 years. The sample (*n* = 127) was larger than previous studies in this area. This RCT found statistically significant improvements in self-regulation and communication for the intervention group only.

### 1.3. The Present Study

This study employs a case-control pre- to post-test intervention design, with an on-task measure of engagement completed for the intervention group. We make four methodological improvements recommended to AAI researchers [4]. Firstly, we adopt measures of symptomology and social functioning used widely in ASD intervention research. This facilitates cross-study comparisons and greater standardisation of AAI studies [1,2,3,4]. Secondly, we include a control group to avoid a major weakness of AAI research [1,4,56], although randomisation of the sample population was not possible. This is because the study was conducted in a real-world school setting, with horse riding sessions set as part of the curriculum. Thirdly, we employ an on-task measure of intervention engagement to examine the relation between within-intervention factors and pre- to post-test outcomes. On-task intervention measures can help reveal which aspects of an intervention influence pre- to post-test change, and studies of AAIs for ASD have tended to neglect using both on-task and pre-post test measures [1]. It should be noted it was not feasible for measures to be completed by blind assessors in this study. Fourthly, measures providing insight into children’s baseline symptoms, concurrent treatments and treatment fidelity were included. Previous studies of AAIs for ASD have tended not to record these factors, which can impact treatment outcomes and the interpretation of findings [4].

### 1.4. Research Questions

(1) Does the horse riding intervention lead to greater change in social functioning, as measured by the CARS2 and the ABC-C, than education as usual?
(2) Do children’s social functioning levels at baseline, as measured by the CARS2 and ABC-C, relate to their level of engagement in the horse riding sessions as measured by the Measurement of Pet Intervention Checklist (MOPI)?
(3) Do children who engage the most in the horse riding sessions, as measured by the MOPI, improve the most from pre-test to post-test?

## 2. Methods

### 2.1. Participants

Twenty-six participants (22 males and 4 females) were recruited from a UK school for children with ASD. Their ages ranged from 6.08–9.33 years (*M* = 7.5, *SD* = 10.57) when the pre-test measures were completed. The intervention group’s mean age was 8.2 years (*SD* = 10.56) and the control group’s was 7 years (*SD* = 3.95). All children had a formal medical diagnosis of ASD carried out by a staff team at the local Social Communication Clinic, which included a clinical psychologist, speech and language therapist and a medically trained paediatrician. Both groups were mainly comprised of low functioning males with a severe level of ASD. Although one participant’s pre-test CARS2 score indicated that they had no or minimal symptoms of ASD, they had been diagnosed with ASD at the age of two years. The majority of participants were “mainly nonverbal” with approximately one third having “limited language”. This was judged by teachers based on their experience observing the children in the classroom. “Mainly nonverbal” indicates that the child can use approximately 5 single words in familiar contexts, but no verbal phrases or sentences. “Limited language” indicates that the child is able to use short phrases in routine contexts, but no spontaneous language.

Using a waiting list design, children were assigned to the intervention or control group condition based on their class membership: two classes were scheduled to have horse riding sessions and two classes were not. Participants in all four classes received Speech and Language Therapy in a whole class format for at least half a day a week, throughout their time at school. Two participants from the control group, and one from the intervention group, also had weekly occupational therapy sessions during the data collection period. Most participants had not taken part in horse riding before, with the exception of four in the intervention group, who had done so more than 2–3 years before the pre-test measures were completed. Children were excluded from the intervention group if they could not wear a helmet, had a known history of treating animals roughly, or feared or disliked animals.

Children with ASD are highly vulnerable and so ethical codes were strictly followed to protect their rights and wellbeing. The welfare of horses was also a key consideration. This was maintained by careful selection of horses for the riding sessions and detailed monitoring of their welfare during the intervention by riding school staff. This research project was approved by the University of Edinburgh Department of Clinical and Health Psychology Research Ethics Committee (reference CLIN280). Parents were sent an information sheet about the aims and nature of the research and were informed that all measures would be collected by teaching staff. Therefore, data would be collected around the children, as opposed to directly from them. Parents also received an opt-out consent form, at the request of the school, and no data was collected about children whose parents returned this form. Given that the horse riding intervention was an established part of the school’s curriculum, parental consent to the horse riding was gained separately to this evaluation study. Every child has the opportunity to participate in horse riding sessions during their time at the school, and children in the control group were given access to the intervention following completion of this study.

### 2.2. Measures

The CARS2 and the ABC-C were completed at pre- and post-test to assess severity of ASD symptoms and challenging behaviours. The CARS2 and the ABC-C have been used by published studies on AAIs for ASD [24,30,32,57]. The MOPI, an observational measure of the child’s engagement during the horse riding, was also completed for participants in the intervention group only. This is the first time the MOPI has been used for a horse riding intervention with children with ASD. All measures were completed by school teaching staff who were familiar with the children.

#### 2.2.1. Childhood Autism Rating Scale, Second Edition (CARS2)

The CARS2 [58] measures the severity of ASD core symptoms on a behavioural rating scale. Its 15 items provide a comprehensive measure of an individual’s functioning that is commonly used to support, but not solely determine, the diagnosis of ASD [59]. The CARS2 standard version (CARS2-ST) is identical to the original CARS and is suitable for low functioning children, thus it was used for most participants. The CARS2 includes a high functioning version (CARS2-HF) which was completed for three participants in the study. Participants were identified as high functioning by their teachers based on their experience of observing them in the classroom. These participants display personal independence in basic activities, such as dressing, toileting and eating. All other participants were assessed using the CARS2 standard version.

An overall CARS2-ST score of 30–36.5 signifies a mild to moderate level of ASD, and a score of 37–60 signifies a severe level. An overall CARS2-HF score of 28–33.5 signifies a mild to moderate level of ASD, and a score of 34 or higher signifies a severe level. Estimates of Cronbach’s alpha for the CARS2-ST (*a* = 0.93) and CARS2-HF (*a* = 0.96) have indicated good internal consistency [58].

#### 2.2.2. Aberrant Behaviour Checklist-Community Edition (ABC-C)

The ABC-C [60] measures a variety of challenging behaviours that can be associated with ASD. It is a 58 item checklist with five subscales: Irritability, Lethargy, Stereotypy, Hyperactivity and Inappropriate Speech. The ABC-C uses a 4-point rating scale (0–3), with higher scores signifying more problematic behaviours. Its subscales stand alone, as the manual does not recommend use of the overall ABC-C score, and each subscale has high internal consistency, recognised validity and sufficient reliability [60]. The ABC-C has been found to be a responsive measure of medication and psychosocial treatment effects [61] and its factor structure is robust and valid in ASD [62]. The ABC-C has been recommended for use with minimally verbal children with ASD [63].

Studies of behavioural problems in ASD have typically used measures intended to support the diagnosis of ASD, such as the CARS2, which do not measure certain associated behaviours of ASD, such as irritability and hyperactivity [64]. The study presented here assessed how core and accompanying features of ASD might change as a result of the intervention, through the CARS2 and the ABC-C respectively.

#### 2.2.3. Measurement of Pet Intervention Checklist (MOPI)

The MOPI [65] is a four item observational measure that assesses participant’s engagement in an AAI, via their attention span, physical movement, verbal communication and compliance. It uses a seven-point Likert scale, denoting no evidence (1) to strong evidence of the behaviour (7), and includes a brief section for qualitative comments about the participant. It is completed after each intervention session for each participant. The MOPI has been used to assess the impact of AAIs involving dogs with severely disabled children [66,67] and adults with intellectual disabilities [68]. Although the MOPI has not been used for a sample of children with ASD, it was chosen in the spirit of replicating measures used by other AAI studies. It is featured in a compendium of established measures used to assess human-animal interactions [69].

### 2.3. Procedure

#### 2.3.1. Pre- and Post-Tests

Pre- and post-tests of the ABC-C and the CARS2 were completed for all participants before and after an approximate 7-week time period. They were completed for each participant by their class teacher based upon their own, and other staff members’, observations of the participant during class in the previous 3–5 days. For the first class, the ABC-C and CARS2 pre-tests were completed the day before their first horse riding session, and the post-tests were completed 1–2 days after their last session. For the second class, the ABC-C and CARS2 pre-tests were completed one week before the first session, and the post-tests were completed 8–9 days after their last session. This ensured that the time periods between the pre- and post-tests were approximately equivalent for both classes.

#### 2.3.2. Horse Riding Intervention

The intervention group consisted of two school classes who attended weekly horse riding sessions. Each session lasted approximately 45 min. Due to timetabling practicalities, the first class was offered seven riding sessions, and the second class was offered five riding sessions.

During the AAI, most children were supervised by a riding instructor who led their horse. The instructors’ training was accredited by the British Horse Society. The instructor and a sidewalker closely monitored the child’s behaviour to ensure their safety on the horse. The sidewalker provided extra support in case the child sat or moved unsteadily during riding. As the intervention progressed, a minority of more independent and capable children no longer required the additional support of the sidewalker, and so this member of staff watched as the child was supervised and led by an instructor. Instructors also carefully monitored the welfare of the horses, as there is a risk that individuals with severe ASD may view and treat animals as objects, rather than sentient beings [37]. Children could not always be paired with the same instructor, but the instructors became familiar with all of the children. All sidewalkers (school teaching staff and volunteers) were very familiar with all of the children.

#### 2.3.3. Horse Riding AAI: Preparing and Mounting (15 min)

At first, children were supported to put on their helmets and boots by teaching staff. When a child was sensitive to wearing their helmet, they were led in sight of the horses and told in an encouraging manner, “Hat on first!”, after which they usually agreed to wear their helmet and so could participate in the session. Children were led to the outdoors or indoors riding arena, depending on weather conditions. One by one, they were assisted by a sidewalker and an instructor to ascend a platform and mount their horse or pony. Each child was paired with a particular horse or pony according to the child’s size, and was kept with the same animal every week as far as was possible, to encourage bonding.

#### 2.3.4. Horse Riding AAI: Riding Skills and Exercises (30 min)

Throughout the session, a lead instructor stood in the centre of the arena and called out instructions for different riding activities: holding the reins, halting, walking and a sitting trot. A rising trot was an additional activity for the more capable children only. Each child was spoken to by their sidewalker and/or instructor throughout the session, who repeated the instructions given by the lead instructor, for example, “Let’s do some trotting now”, commented on the activities being fun and praised the child for appropriate behaviours.

After the riding activities, whilst sitting on their horses, children did some stretching exercises for a few min. Then, each child was supported to dismount their horse by an instructor and sidewalker. The latter modelled petting the horse, thanking and saying goodbye to the instructor and horse, and encouraged the child to do the same. Outside of the riding arena, children were supported to remove their helmets and boots.

#### 2.3.5. Completion of the MOPI

Teaching staff completed the MOPI at the end of each horse riding session for the participant they had accompanied horse riding. This was the same child each week as far as was possible to encourage rating consistency, although sometimes it was not possible due to staff or child absences. Teaching staff discussed how they decided on their MOPI ratings, which enabled more consistent ratings across staff. They asked for one another’s observations of their allocated child if they felt they had missed any noteworthy behaviours, which facilitated well-informed ratings. In the case of children who rode with just the support of an instructor, the staff member who had observed their session asked the relevant instructor for further detail on the child’s behaviour in order to complete the MOPI.

The MOPI’s qualitative comments provided detail on the extent of each child’s participation and enabled overall treatment fidelity data to be calculated. Although most children participated in each full riding session, occasionally a child would not ride their horse at all. Usually this was accounted for by a child’s agitated mood. Such cases were recorded as infidelity to the treatment for that session. Sometimes a child grew restless before the session was over, and was taken off of the horse approximately 5 min early. These instances were *not* interpreted as treatment infidelity, because the child had participated in most of the session and it is appreciated that children with ASD tend to have high rates of inattention and impulsivity [70,71]. Therefore, full participation in every riding session would be an unrealistic expectation. Whenever a child did not ride, they sat with a member of staff in the spectators’ section of the arena to watch the other children riding. The instructors led the child’s horse in front of the spectating child, at a safe distance, to encourage greater familiarity and comfort with the animal. Subsequently, one child was able to be supported in leading their horse around the arena, although they did not actually ride it.

### 2.4. Analyses

Pre- to post-test changes were statistically tested to answer Research Question 1. The pre- and post-test scores for the CARS2 and the Hyperactivity and Irritability subscales of the ABC-C met the parametric assumptions of normality and homogeneity of variance of a mixed analysis of variance (ANOVA). Mixed ANOVAs were conducted to investigate whether there was a significant pre- to post-test improvement for the intervention group compared to the control group. Significant interactions were then followed up with paired-sample and independent *t*-tests. The pre- and post-test scores of the Lethargy, Inappropriate Speech and Stereotypy subscales of the ABC-C did not meet the parametric assumptions of a mixed ANOVA. Therefore, Mann Whitney U tests were used to assess between-group differences in pre- to post-test change, and Wilcoxon signed-rank tests to assess within-group differences in pre- to post-test scores.

The MOPI’s relationship with baseline ABC-C and CARS2 scores (Research Question 2) was investigated using Pearson’s correlations, because these pre-test scores had met parametric assumptions. Then, the MOPI’s relationship with the test measures which significantly improved from pre- to post-test: the CARS2 and Hyperactivity subscale, was investigated (Research Question 3). Spearman-ranked correlation coefficients were used because pre- to post-test change scores had not meet parametric assumptions.

## 3. Results

### 3.1. Data Preparation

Although 26 participants had been recruited, two children in the intervention group were excluded from data analysis because they did not ride their horses at all. The remaining 10 children in the intervention group had a mean treatment fidelity rating of 0.79 (*SD* = 0.27), with further details shown in Table 1. This table also shows that at baseline, the intervention and control groups did not significantly differ in terms of: gender, functioning level, verbal ability, severity of ASD symptoms and P scale levels in English, Maths and Science [72]. A significant difference was found between the groups’ mean ages.

Reliability analyses showed that the MOPI’s overall score and the pre- and post-test scores of the ABC-C subscales had good internal reliability; all Cronbach’s alpha scores were above 0.7 with the majority greater than 0.8, apart from the Lethargy subscale at pre-test, for which *a* = 0.662. Subsequently, for every participant, pre- to post-test change scores were calculated for each of the ABC-C subscales and the CARS2, by subtracting a pre-test score from its associated post-test score.

Shapiro-Wilks, Levene’s test and z-scores of skewness and kurtosis were then calculated for each test measure’s pre-test, post-test and change score to determine whether parametric assumptions were met for subsequent analyses.

### 3.2. Research Question 1: Effectiveness of the Horse Riding Intervention

Means and standard deviations for all pre-test, post-test and change scores and interaction effects are reported in Table 2. Before the mixed ANOVAs were conducted, independent samples *t*-tests revealed no significant between-group differences in baseline scores for Hyperactivity, *t*(22) = 1.171, *p* = 0.254, *r* = 0.242; Irritability *t*(22) = −0.581, *p* = 0.567, *r* = 0.123 and the CARS2, *t*(22) = 0.575, *p* = 0.571, *r* = 0.122.

**Figure 1:**
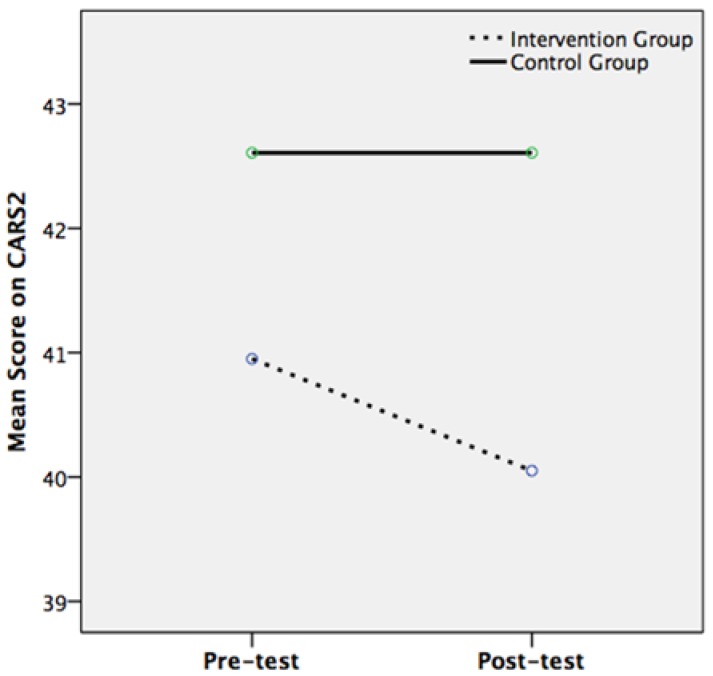
Mean Childhood Autism Rating Scale, Second Edition (CARS2) scores at pre- and post-test for the intervention group (*n* = 10) and control group (*n* = 14).

The mixed ANOVA for the CARS2 found a significant main effect of time (*F*(1, 22) = 7.219, *p* = 0.01, *r* = 0.5) but a non-significant main effect of intervention condition (*F*(1, 22) = 0.549, *p* = 0.466, *r* = 0.156). There was a significant interaction effect between pre- to post-test change in CARS2 scores and intervention condition (*F*(1, 22) = 7.219, *p* = 0.013, *r* = 0.5). Whilst the control group had the same mean CARS2 scores at pre-test and post-test (42.61), the intervention group’s mean score was significantly lower at post-test (40.05) than at pre-test (40.95) (Figure 1). Follow-up tests were non-significant, apart from a marginally significant difference between CARS2 scores at pre- and post-test for the intervention group only (*t*(9) = 2.250, *p* = 0.051, *r* = 0.6). This may be explained by the ANOVA’s power being limited by a relatively small sample size [73]. Nonetheless, the CARS2 pre- and post-test data had met the assumptions of the mixed ANOVA, which has the advantage of limiting the likelihood of a Type 1 error, in comparison to using just *t*-tests [74].

**Figure 2:**
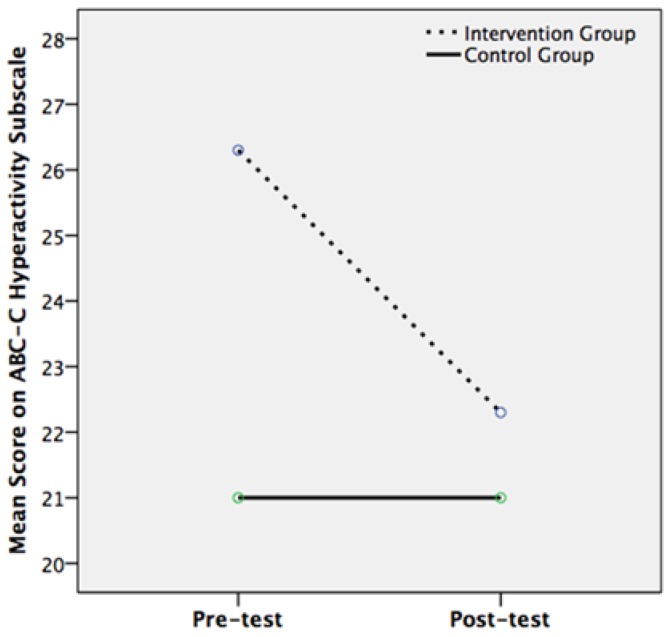
Mean Hyperactivity scores on the ABC-C at pre- and post-test for the intervention group (*n* = 10) and control group (*n* = 14).

For the Hyperactivity subscale of the ABC-C, there was a significant main effect of time (*F*(1, 22) = 8.084, *p* = 0.009, *r* = 0.518) but a non-significant main effect of intervention condition (*F*(1, 22) = 0.568, *p* = 0.459, *r* = 0.159). There was a significant interaction effect between pre- to post-test change in Hyperactivity scores and the participant’s condition (*F*(1, 22) = 8.084, *p* = 0.009, *r* = 0.518). This signifies a large effect size, as it is above Cohen’s benchmark of 0.5. Whilst the control group had the same mean Hyperactivity scores at pre- and post-test (21), the intervention group’s mean score was significantly lower at post-test (22.3) than at pre-test (26.3) (Figure 2). Follow-up independent and paired-samples *t*-tests were carried out to probe the interaction. A significant difference was found between pre- and post-test scores for the intervention group only (*t*(9) = 2.4, *p* = 0.040, *r* = 0.625), which denotes a large effect size. The mixed ANOVA for the Irritability subscale of the ABC-C found no significant main or interaction effects (Table 2). Wilcoxon signed rank and Mann Whitney U tests showed no significant within-group, between-groups or interaction effects for the Lethargy, Inappropriate Speech and Stereotypy subscales of the ABC-C.

### 3.3. Research Question 2: Symptoms and Behaviour at Baseline and Intervention Engagement

Analyses were conducted to explore whether baseline scores of social functioning influenced how much children engaged with the riding sessions, as measured by the MOPI. Pearson’s correlations were computed between MOPI scores and the mean pre-test scores of the CARS2 and each of the ABC-C subscales. More severe ASD symptoms at baseline were related to less overall engagement with the riding sessions and fewer instances of verbal communication. This was shown by the significant negative correlation between pre-test CARS2 scores and overall MOPI scores (*r* = −0.664; *p* = 0.036) and between pre-test CARS2 scores and mean communication scores on the MOPI (*r* = −0.678; *p* = 0.031). A greater level of hyperactivity at baseline was also associated with lower engagement in the intervention; pre-test Hyperactivity scores had a significant negative correlation with mean attention (*r* = −0.662, *p* = 0.037) and compliance scores on the MOPI (*r* = −0.632, *p* = 0.05). These findings support the MOPI’s reliability, as one would expect a participant with more severe ASD symptoms to present a higher degree of communication difficulties, and a more hyperactive participant to have lower attention and compliance scores. No significant Spearman’s correlations were found between change and baseline scores on the CARS2, nor between change and baseline scores on the Hyperactivity subscale of the ABC-C. This indicated that there was no significant association between pre-test to post-test improvement and participants’ social functioning level at baseline.

### 3.4. Research Question 3: Intervention Engagement and Intervention Effectiveness

**Figure 3:**
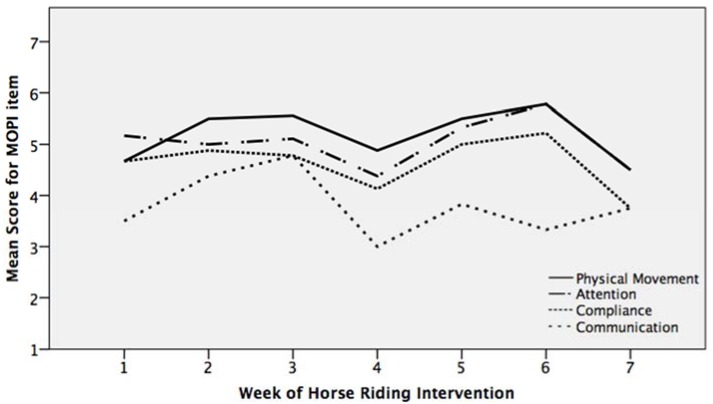
Mean Measurement of Pet Intervention Checklist (MOPI) item scores across the intervention period for the intervention group (*n* = 10).

Participants’ engagement with the horse riding sessions was measured by the MOPI. The intervention group had their highest mean MOPI scores for physical movement, followed by attention, compliance, and lastly communication (Figure 3). On the MOPI scale, ratings of 2 and above indicated positive engagement in the intervention, and the intervention group’s mean MOPI item scores ranged between 4 and 6, indicating a very positive level of engagement. The exception to this was the mean verbal communication score. This can be explained by the participants being predominantly nonverbal, which limited their ability to score highly on this item. Participants’ mean MOPI item scores decreased during the fourth session (Figure 3), which occurred the day after a bank holiday for 70% of the intervention group. The teaching staff reported that participants were less focused during this session as a result of their disrupted weekly routine: “It was difficult for the child to concentrate after the bank holiday. They kept taking their helmet off and flicking their fingers in the air.”

The MOPI also provided a range of qualitative data on children’s engagement with the horse riding intervention. One comment noted that the child said “...‘Walk on’ and ‘Go faster!’, laughed, smiled and demonstrated excitement” as they rode their horse. Another participant “…requested his boots, was really happy and eager to get on the horse, used some riding terminology and laughed when the horse trotted.” Many children became more engaged as the intervention progressed, for example, during one participant’s first riding session they “…threw their boots and helmet ... (and showed) signs of anxiety—lots of rapid movements of their hands and head.” Yet, by the fourth session, this participant was “…happy when riding and trotting, waved “hello” when called by staff (and was) restless when (the) horse stopped.” In other instances, the qualitative data helped explain a child’s lack of apparent engagement in the riding session. For example, in one session a participant “…got on the horse twice for a few seconds only…and had to be taken off.” The MOPI rater hypothesised that sensory issues had prevented this child’s participation, because they made progress after they could wear their helmet without any difficulties.

Within this context of positive engagement, there was no evidence that participants’ engagement levels, as measured by the MOPI’s numerical ratings, related to their pre- to post-test improvement in social functioning. No significant correlations were found between overall MOPI scores and CARS2 change scores, nor between CARS2 change scores and any of the four MOPI items. There were also no significant correlations between Hyperactivity change scores and the four MOPI items. There was only one significant correlation between Hyperactivity change scores and the mean overall MOPI score (*r* = 0.671, *p* = 0.034), which would imply that children with higher MOPI scores had the *least* improvement in hyperactivity. However, given that only 1 out of 10 possible correlations in this analysis was significant, it is potentially spurious and should be treated with caution. The MOPI had high internal consistency, with Cronbach’s alpha scores for each week of the intervention ranging from 0.78 to 0.95.

## 4. Discussion

The horse riding intervention led to a greater change in social functioning than education as usual (Research Question 1). At post-test, there was a significant reduction in the severity of ASD symptoms, as measured by the CARS2 and the Hyperactivity subscale of the ABC-C, for the intervention group only. No significant changes were found in participants’ levels of lethargy, irritability, stereotypy nor inappropriate speech after the intervention period. Therefore, the current study suggests that horse riding interventions may be beneficial to low functioning nonverbal children with severe ASD, aged 6–9 years, for aspects of their social functioning.

The current study found significant improvements in social functioning after a shorter intervention period than other AAI studies that have used the CARS2 and ABC-C. Kern et al. (2011) [24] found that the severity of ASD symptoms was significantly reduced after three months of horse riding. Most participants in Kern et al.’s (2011) study had a severe level of ASD, making them comparable to the current study’s sample, although Kern et al. (2011) did not use a control group, which makes their findings less robust.

Regarding the ABC-C, Gabriels et al. (2015) [57] found significant reductions in hyperactivity and irritability after 10 weeks of horse riding. By contrast, the current study suggested that changes in hyperactivity can occur after just 5–7 weeks. This would require less funding from parents and/or educational or therapeutic institutions, although further research is needed to test whether this finding is replicated in different samples. Gabriels et al.’s (2015) [57] participants were children or adolescents; however, their mean age was 10.2, making it comparable to the current study’s mean age. It should be noted that Gabriels et al.’s (2012) [30] pilot study found significant improvements in all of the ABC-C subscales, excluding Inappropriate Speech, after a 10-week intervention period. This suggests that a longer intervention period may lead to more extensive improvements in social functioning as measured by the ABC-C, than those reported by the current study.

Regarding the second research question, more severe ASD symptoms at baseline were related to lower overall engagement in the intervention and lower levels of verbal communication, as measured by the MOPI. In addition, greater hyperactivity at baseline was associated with lower attention and compliance scores on the MOPI. These findings suggest that more severe ASD symptoms and hyperactivity at baseline may have limited participants’ ability to engage, or to behaviourally demonstrate engagement, in the riding tasks. It could be speculated that participants with more severe ASD symptoms at baseline had a limited capacity to achieve high MOPI scores, and that this may not have been the case for participants with less severe ASD symptoms. Nonetheless, these children showed significant improvements in social functioning as a result of the intervention.

There was no evidence that participants’ level of engagement in the AAI related to their pre- to post-test improvement in social functioning (Research Question 3). Overall, engagement in the intervention was good; mean MOPI item scores ranged from 3 to 6 across the intervention period and ratings of 2 and above signified positive engagement. Thus, the current study found that even if participants did not achieve high levels of engagement as measured by the MOPI, they still benefitted from the AAI through reduced hyperactivity and ASD symptoms from pre- to post-test. Additionally, having more severe ASD symptoms at baseline, which was associated with lower MOPI scores, did not impinge upon a participant’s pre- to post-test improvement in social functioning. This was shown by baseline CARS2 and hyperactivity scores being uncorrelated to their respective pre- to post-test change scores.

Based on these findings, it seems that the extent of compliance and engagement with riding tasks did not influence the effectiveness of AAI in this study. Rather, the general experience of riding, such as touching the horse or moving to a rocking rhythm, may have helped reduce ASD symptomology and hyperactivity. This is in line with existing literature on the physiological benefits of AAIs referred to in the introduction, and can help inform research on possible mechanisms of AAIs for ASD. Similar to other AAI studies, our findings provide more evidence of an AAI’s efficacy, rather than the reasons why it might be efficacious [55,56].

### 4.1. Implications

The intervention resulted in measurable gains in important aspects of social functioning for children with ASD. Hyperactive behaviour is a common symptom for children with ASD [71], and reduced hyperactivity is associated with improved self-regulation. Thus, the AAI in the present study may have enabled children to function more effectively at home, school and in other public places [30]. Furthermore, improved social functioning may have been beneficial to children’s caregivers and is an area for future research. It is particularly pertinent given that managing the behaviours of a child with ASD is associated with higher stress levels for caregivers, than is the case when caring for a child with other special needs [75].

A number of practical implications emerged from the study concerning how to effectively engage children with ASD in AAIs. For children who observed the session because they were hesitant to mount their horses, gentle attempts were made to familiarise them with the animals. Riding instructors showed the horses to the children from a safe distance. This led to one participant agreeing to hold an instructor’s hand to lead the horse around the arena, and had the intervention been longer, may have led to the child riding the horse.

Yet, particular animal noises or smells may be distressing for some children with ASD [37], which might explain why two participants chose not to ride their horses at all. It is important to play close attention to children’s reactions to the animals, and to never pressurise a child to be involved in an AAI. This avoids causing the child or the animal any distress. In addition, adults who accompany children with ASD on AAIs should be aware that sessions following disrupted routines may lead to difficult behaviour. This was demonstrated by the intervention group’s mean MOPI item scores declining during the fourth riding session, which followed a bank holiday for most participants and altered their usual weekly routine. The children’s reaction can be partly understood in terms of a common symptom of ASD, aversion to change [7].

### 4.2. Strengths and Methodological Limitations

The current study was guided by the need for greater methodological rigour, standardisation and cross-study comparisons across AAI studies [1,2,3,4]. To help address the question of whether AAIs for ASD are efficacious, this study utilised: a control group, a relatively large homogenous sample, and multiple assessment measures; all features uncommon in existing research on AAIs in ASD [3]. Measures covered a broad range of ASD behaviours: the CARS2 assessed core features related to ASD diagnosis, and the ABC-C measured accompanying problem behaviours not included in diagnosis [64]. Also, the current study reported treatment fidelity in a standardised way, which studies on AATs in youth have rarely done [3]. Overall, participation in the riding sessions was good, with 70% of participants who completed the study riding their horse for 75–100% of available sessions. This strengthens the study’s internal validity by providing supporting evidence that the improvements in social functioning were caused by the intervention itself. Furthermore, the two participants who did not ride their horses at all were excluded from data analysis to ensure valid comparisons were made between control and intervention groups.

As a case-control study, there was no randomised assignment of participants to groups, which would have reduced possible systematic differences from influencing group differences. However, this was not possible because classes attended horse riding as part of the school curriculum. Instead, the current study used well-matched intervention and waiting list control groups in terms of: gender, severity of ASD symptoms, verbal ability, functioning level and approaches to education. By controlling for group differences [3,76], this method creates a more homogenous sample that is more likely to find evidence of a treatment effect, if one exists [55]. Fewer group differences decrease the likelihood of variation in outcome measures being explained by factors other than participation in the intervention. Given the wide heterogeneity in ASD’s severity and symptoms, matching such a sample can be difficult to achieve, and is a relatively unique strength of the current study. This is suggested by none of the studies in a key systematic review of equine-assisted interventions using a matching technique [2].

A limitation of the current study is that no longer-term follow-up data were collected, which could have investigated whether the reductions in ASD severity and hyperactivity were maintained. The side walker and trained riding instructor may have been confounding variables influencing the reduction in ASD symptoms. It is difficult to assess whether the presence of the animal, the human, or a combination of both, influenced the intervention’s efficacy [4]. Also, it was not possible to use blind-raters, a common methodological limitation in research on AAIs for ASD [1,4]. This presents the risk of a possible expectancy effect by the teaching staff who filled in the measures. Yet, to facilitate accurate assessments, the pre- to post-test measures needed to be completed by adults who closely observed and were familiar with children’s behaviour.

The MOPI usefully measured engagement in the AAI, but could be refined to be better-suited to participants with ASD. Firstly, a nonverbal communication item could be added to capture the main way participants in the current study communicated with their side walker and instructor. Although nonverbal communication could be recorded in the MOPI’s qualitative section, this data could not be incorporated into statistical analyses. Secondly, a participant’s behaviour could not always be rated straightforwardly as compliant or non-compliant. For example, some children misinterpreted the instruction to “stop” (their horses) as the end of the session, and consequently attempted to remove their helmets and behaved in an unsettled manner. This was also rated as non-compliant, but had been unintentionally so. Sometimes a child performed a riding skill when they had not been instructed to do so. This was rated as non-compliant, but the teaching staff recorded that in a sense the child had acted progressively by practising a new skill. As the sample was predominantly low functioning, at times children may have been unable to respond to instructions immediately. Thus, the compliance item should be refined to refer to intentional non-compliance to the intervention, to make it more straightforward to complete.

It should also be noted that 3 of the 5 ABC-C subscales needed to be analysed using non-parametric tests, which were unable to detect potential interaction effects. They also neglected the magnitiude of differences between scores because the data was ranked. Furthermore, whilst the current study had a larger sample than most AAI studies for ASD (64% of studies reviewed by O’Haire (2013) [4] had a sample less than 12), it was still relatively small for ANOVA analyses. A larger sample would have had more statistical power [73], although recruiting ASD populations can be difficult. In addition, it would be valuable to investigate whether these results are replicated for adolescents with low functioning ASD. If future research studies achieve sufficiently large samples, randomised allocation to experimental and control conditions is recommended to assess an AAI’s efficacy more robustly. In future, researchers should also aim to use: multiple outcome measures replicated from existing studies to enable cross-study comparisons; blind raters; and longer-term follow-up data to assess the longevity of AAI effects.

## 5. Conclusions

The current study demonstrated a significant reduction in hyperactivity and ASD symptoms for the intervention group only, and for a shorter intervention period than existing AAI studies that also used the CARS2 and ABC-C as assessment tools. Greater improvements in social functioning were unrelated to level of engagement with the horse riding, however, the MOPI revealed that overall engagement was very positive. This relatively large and well-matched between-groups design study, which used an innovative on-task measure and established measures of social functioning, provides a valuable contribution to research on AAIs for ASD.

## Figures and Tables

**Table 1:** Characteristics of Participants Who Completed the Study.

Pre-Test Characteristic	Intervention Group (*n* = 10)	Control Group (*n* = 14)	Total Sample (*n* = 24)
Mean age in years (*SD*)	7.96 (0.78)	6.97 (0.33)	7.38 (0.74)
Gender			
Male	9	12	21
Female	1	2	3
Functioning level ^a^			
Low	10	11	21
High	0	3	3
Verbal ability ^a^			
Mainly nonverbal	6	10	16
Limited language	4	4	8
Severity of ASD symptoms ^b^			
None-minimal	0	1	1
Mild-moderate	3	1	4
Severe	7	12	19
Mean CARS2 score ^c^ (*SD*)	40.95 (6.07)	42.61 (7.52)	41.92 (6.86)
Median P scale levels ^d^			
English	5	4.5	5
Maths	7	5	6
Science	4.5	5	5
Treatment fidelity rating ^e^			
Percentage of sessions participants rode in			
75–100%	7	N/A	N/A
50–74%	1	N/A	N/A
25–49%	2	N/A	N/A
0–24%	0	N/A	N/A

Note: Fisher’s exact tests were used for categorical variables. At baseline there were no significant differences between intervention and control groups in: gender (*p* = 1), functioning level (*p* = 0.239), verbal ability (*p* = 0.673) and severity of ASD symptoms (*p* = 2.72). Independent-samples *t*-tests were used to compare mean pre-test CARS2 scores and mean age. There was no significant difference between pre-test CARS2 scores (*p* = 0.571). A significant difference was found between the groups’ mean age (*p* = 0.003). ^a^ Functioning level and verbal ability were reported by each participant’s teacher, based on their experience observing and interacting with each child in the classroom. Participants were identified as high functioning if they display personal independence in basic activities, such as dressing, toileting and eating. “Mainly nonverbal” means the child can use approximately 5 single words in familiar contexts, but no verbal phrases or sentences. “Limited language” means the child is able to use short phrases in routine contexts, but no spontaneous language. These non-clinical definitions are shared and understood by staff; ^b^ Severity of ASD symptoms was based upon participants’ pre-test CARS2 scores; ^c^ The mean CARS2 scores in the table indicate a severe level of ASD on both the low functioning and high functioning versions of the assessment measure; ^d^ Mann-Whitney U tests found no significant differences between groups’ P scale levels for English, Maths and Science. P scales are a national assessment tool for children with special educational needs who are working below National Curriculum levels [72]. A child’s P scale levels were based on teachers’ professional judgements. They compare the child’s work and performance with the P scale level descriptors. The school carries out moderation exercises several times a year to ensure that P scale level judgements are fair and correct, as far as is possible; ^e^ Treatment fidelity ratings were collected for the intervention group only, based on the qualitative section of the MOPI. Treatment infidelity was reported for an individual each time they did not ride their horse at all, during a session. Cases in which a child grew restless and was taken off their horse approximately 5 min before the end of the session were not interpreted as treatment infidelity. This is because the child participated in most of the session.

**Table 2:** Analysis of efficacy of horse riding intervention compared to an education as usual control.

Test Measure	Intervention Group (*n* = 10)						Control Group (*n* = 14)						Interaction ^a^		
	Pre		Post		Pre-Post Change ^b^		Pre		Post		Pre-Post Change ^b^				
	*M*	*SD*	*M*	*SD*	*M*	*SEM*	*M*	*SD*	*M*	*SD*	*M*	*SEM*	*F(1, 22)*	*p*	*ES*
CARS2 Score	40.95	6.07	40.05	5.57	−0.9	0.4	42.61	7.52	42.61	7.52	0	0	7.219	0.013 *	0.5
ABC-C															
Hyperactivity	26.30	10.73	22.30	9.67	−4	1.68	21	11.07	21	11	0	0.15	8.084	0.009 **	0.518
Irritability	20.20	8.78	18.90	7.58	−1.3	1.19	22.50	10.08	22.50	9.83	0	0.26	1.548	0.227	0.256
Lethargy	14.60	3.86	14.90	3.78	0.3	0.9	14.43	5.76	14.14	6.40	−0.3	0.37			
Stereotypy	10	4.74	10.50	3.69	0.5	1.16	7.71	6.09	7.79	6.22	0.08	0.13		N/A	
Inappropriate Speech	3.40	3.89	3.60	3.92	0.2	0.39	2.93	3.56	3.07	3.56	0.14	0.18		N/A	

^a^ The parametric requirements of a mixed ANOVA were only met by the pre- and post-test scores on the CARS2, Hyperactivity and Irritability subscales of the ABC-C.; ^b^ Pre- to post-test change scores were calculated as: “post-test score” – “pre-test score”. Therefore, a negative change score denotes a reduction in symptoms from pre- to post-test. By contrast, a positive change score denotes a worsening of symptoms from pre- to post-test; * denotes that *p* < 0.05 and ** denotes that *p* < 0.01 for testing the mixed ANOVA.
